# Numerical Investigation of Transonic Flow-Induced Spontaneous Condensation in Micro-Ejector Nozzles

**DOI:** 10.3390/mi14061260

**Published:** 2023-06-16

**Authors:** Yu Han, Xiaodong Wang, Wei Wang, Yuan Xien Lee, Ao Li

**Affiliations:** 1 School of Mechanical Engineering and Automation, Northeastern University, Shenyang 110819, China; yuhan.neu@gmail.com; 2 School of Mechanical and Electrical Engineering, Chuzhou University, Chuzhou 239004, China; 3 School of Mechanical and Manufacturing Engineering, University of New South Wales, Sydney, NSW 2052, Australia; wei.wang15@unsw.edu.au (W.W.); leeyuanxien@gmail.com (Y.X.L.)

**Keywords:** micro-ejector, nozzle, transonic flow, wet steam, spontaneous condensation

## Abstract

Micro-cooling systems are compact refrigeration systems widely applicable in microchemical analysis, biomedicine, and microelectromechanical systems (MEMS). These systems rely on the use of micro-ejectors to achieve precise, fast, and reliable flow and temperature control. However, the efficiency of micro-cooling systems is hindered by spontaneous condensation occurring downstream of the nozzle throat and within the nozzle itself, impacting the performance of the micro-ejector. A micro-scale ejector mathematical model describing wet steam flow was simulated to investigate the steam condensation phenomenon and its influence on flow, incorporating equations for liquid phase mass fraction and droplet number density transfer. The simulation results of wet vapor flow and ideal gas flow were compared and analyzed. The findings revealed that the pressure at the micro-nozzle outlet exceeded predictions based on the ideal gas assumption, while the velocity fell below it. These discrepancies indicated that condensation of the working fluid reduces the pumping capacity and the efficiency of the micro-cooling system. Furthermore, simulations explored the impact of inlet pressure and temperature conditions on spontaneous condensation within the nozzle. The results demonstrated that the properties of the working fluid directly influence transonic flow condensation, underscoring the importance of selecting appropriate working fluid parameters for nozzle design to ensure nozzle stability and optimal micro-ejector operation.

## 1. Introduction

**Figure 1:**
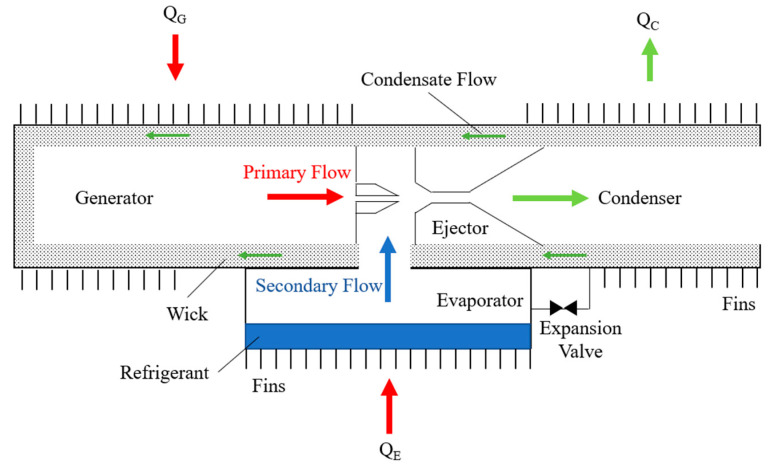
The schematic diagram of the refrigeration system.

Micro-ejector is a miniature device implementing the nozzle principle, mainly used for gas pressurization, vacuum processing, and liquid spraying. Similar to other nozzles, the micro-ejector reduces the pressure of steam by increasing the velocity of the fluid to achieve gas pressurization and vacuum treatment. Micro-ejectors are often employed in micro-cooling systems, which are able to suppress temperatures in limited space, thus suitable for micro-electronic device cooling, micro-sensor cooling and other applications [1]. The micro-ejector plays a vital role in the micro-cooling system, where it serves as the refrigerant injector that injects refrigerants to the micro-cooling chips or other micro-cooling devices to achieve refrigeration. In addition, micro-ejectors can be used for heat recovery to dissipate the heat from the micro-refrigeration device to the surrounding. The micro-ejector can be used as the control device in the micro-cooling system to regulate the airflow and temperature [2]. With precise adjustment of parametric operational conditions of the micro-cooling system, it is possible to achieve a more accurate and efficient cooling effect. Concurrently, micro-ejectors possess the advantages of small structure space and low energy consumption, which are pertinent for micro-refrigeration devices and applications. A heat pipe/injector cooling system consisting of a micro-ejector, condenser, capillary pump, generator, evaporator and throttle valve, is proposed based on the combination of the heat pipe and the micro-ejector [3]. Figure 1 shows the schematic diagram of the cooling system. Contingent upon the practical experiment, a capillary pump replaces the electric circulation pump in a conventional ejector cooling system.

In the past, larger jet-based propulsion systems, such as those operating in the rocket-jet cycle, have been built and operated in wide scales [2]. After the 1960s, ejector systems were scaled down to the microscale domain as a means of entraining and delivering gases for applications other than propulsion. Micro-ejectors provide a unique way to entrain relatively large amounts of air without any moving parts, and this air, mixed with fuel, can provide the necessary fuel–air mixture to power a micro-combustion system [4]. Micro-jet and micro-combustion systems can together form the following components of a unique air-breathing propulsion system [5]. Micro-ejectors can generate sufficient suction to meet the mass flow and power consumption requirements of micro-engines, allowing them to replace high-speed micro-pumping turbomachinery [6]. The microscale ejector allows microscale engines and rockets to meet pumping requirements and achieve heating requirements without high-speed microscale turbomachinery. Hou et al. [7] investigated a high-powered thermal bubble micro-jet with an induction heater to increase liquid delivery speed. This new micro-ejector can also be used for micro-scale liquid delivery, micro-drug injection, and 3D printing. After decades of micro-jet research more for toner manipulation and inkjet printing, Ben Hsieh et al. [8]. developed a supersonic micro-jet device for transdermal drug delivery, administering vaccines in a safe and cost-effective manner while maximizing efficacy. Fan et al. [9] proposed a micro-ejector with a large displacement ratio to provide a fuel–air mixture for a catalytic burner. The operating conditions and geometrical parameters of the ejector were systematically varied and their effect on the volumetric flow rate ratio was investigated experimentally.

Portable cooling and air conditioning systems with high cooling capacity and low power consumption are heavily demanded in various fields, such as electronics, transportation, and medicine. Current research trends lie in the development of micro refrigeration systems with miniature ejectors to satisfy the requirements of higher cooling capacity, portability, and energy efficiency [1]. It emerged as a promising alternative to conventional compressors for miniature refrigeration systems due to their low energy consumption, small size, and ease of integration. The performance of micro-ejectors is critical to achieving effective cooling and heating, and researchers have conducted various investigations to improve their efficiency and optimize their design [10].

Other researchers have focused on understanding the underlying flow mechanisms inside the nozzle that affect the performance of micro-ejectors in miniature refrigeration systems. The phenomenon of spontaneous condensation of steam flowing across the speed of sound is widespread inside miniature fuel cell/fuel reformer systems [11], in the MEMS steam locomotive power generation cycle [12] and in the chemical industry. Spontaneous condensation from the transonic flow within a steam micro-ejector is mainly concentrated downstream of the throat inside the nozzle [13,14]. The spontaneous condensation process of steam occurs in many electrical machinery and engineering equipment, such as supersonic separators, steam turbines and ejectors. The spontaneous condensation of working steams inside the nozzle reduces the efficiency of the nozzle itself and also has a negative impact on the priming of the pumped fluid [15]. Therefore, the study of the spontaneous condensation phenomenon inside the nozzle of a steam jet pump with transonic flow has important scientific significance and application background.

In the practical application of engineering, the nozzle and the body of the ejector are separate, and this structure can make the whole ejector system relatively complex. The primary fluid is accelerated and depressurized inside the nozzle to form a low-pressure area at the nozzle outlet, which sucks the working fluid through the pressure difference before entering the ejector. The flow conditions inside the nozzle have an important impact on improving the pumping performance of the ejector. The intrinsic connection between the condensation behavior of steam and the throat radius of the nozzle, the expansion angle of the evanescent section of the nozzle and the length of the evanescent section of the nozzle were investigated by Li et al. [6]. The results showed that the spontaneous condensation behavior of steam was suppressed by changing the geometry of the nozzle to improve the operating performance of the ejector. Jiang et al. [16] determined a mathematical model for the supersonic condensation process of ternary mixtures in nozzles based on the simulation results of the supersonic condensation process of binary mixtures. The flow characteristics and condensation mechanism of ternary mixtures in supersonic flow were developed. Zhang et al. [17] experimentally and numerically investigated the effect of inlet steam superheat when a steam condensate stream passes through an IWSEP (International Wet Steam Experimental Program) nozzle. The static pressure on the nozzle wall and the liquid phase characteristics at three locations along the nozzle centerline were measured. Sahami et al. [18] investigated the effect of the condensation phenomenon on the propulsion performance of cold gas thrusters under real gas conditions with different expansion ratios and nozzle inlet pressures. Wang et al. [19] used a wet vapor model to investigate the relationship between steam superheat and nonequilibrium condensation in the primary nozzle of an injector. The nonequilibrium condensation process from the homogeneous nucleation to the droplet growth stage and the effect of superheat on the condensation shock wave and total entropy were investigated. Yin et al. [20] studied the condensation flow model of water vapor and investigated the effect of back pressure on the supersonic condensation characteristics of water vapor. The results of the study show that under the action of the shock wave, the condensed droplets evaporate again, and then the droplets undergo secondary condensation due to the cooling effect of the nozzle.

The converging-diverging nozzle is geometrically shaped in such a way that it best represents transonic flow, i.e., subsonic and supersonic flow. The nonequilibrium process of spontaneous condensation has a significant impact on the transonic flow field [21,22,23]. In this work, numerical simulations are used by introducing the liquid phase mass fraction and droplet number transfer equations. The spontaneous condensation of steam in a two-dimensional nozzle is simulated. The flow inside the nozzle under wet steam and ideal gas conditions, as well as the effect on the performance of the injector is discussed. The analysis of the spontaneous condensation of the transonic wet steam two-phase flow was found to have an effect on the flow state inside the nozzle. It was found that the simulations performed by applying an ideal gas differed somewhat from the actual flow, leading to the selection of a fluid state that better reflects the flow characteristics. The effect of working steam parameters on spontaneous condensation is investigated, which, in turn, provides a basis for assessing the effectiveness of the steam spontaneous condensation phenomenon on the overall ejector flow process.

## 2. Numerical Solution Method

The Euler–Euler two-flow model was used to study the law of motion of the primary flow in the nozzle. On this basis, two-phase continuity, momentum and energy control were achieved by establishing the heat and mass transfer equations between the gas and liquid phases, in addition to establishing two additional liquid phase mass ratio transfer equations and droplet number distributions.

**Figure 2:**
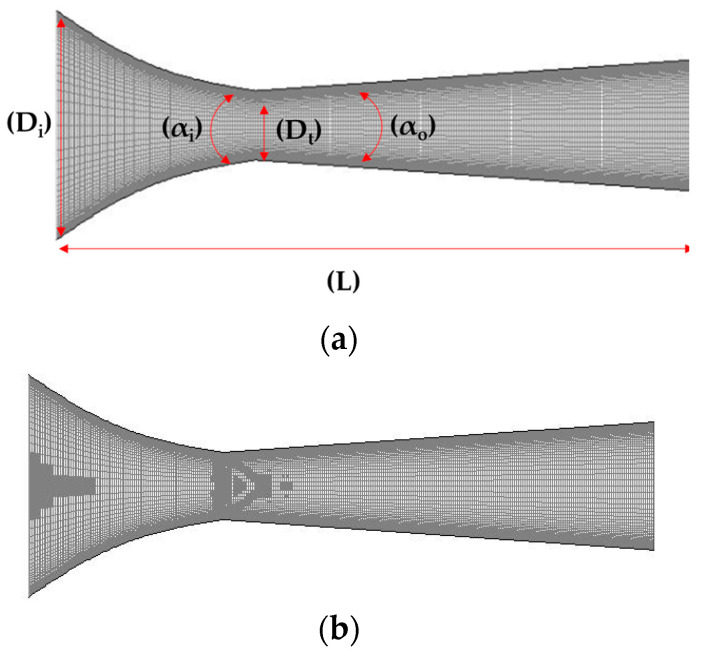
The structured grid of micro-ejector. (**a**) original mesh; (**b**) structure mesh.

The specific geometric parameters of the nozzle are shown in Table 1. It has been shown that the simulation results obtained from the 3D geometric model are very similar to those obtained from the 2D geometric model [24,25]. Therefore, in this study, only the 2D geometric model is structured and meshed, and the partitioned meshing technique (multi-block technique) is used to encrypt the regions where important flow field structures may occur. The initial total number of grid cells is 10,805 (see Figure 2a), and then the grid is locally processed via the gradient adaptive technique, and the final total number of grid cells is 14,037 (see Figure 2b), which establishes the grid independent of the algorithm results.

Employing the realizable *k*-*ε* turbulence model based on the Boussinesq assumption [26], the turbulent kinetic energy *k*, turbulent energy dissipation rate *e*, equation can be expressed as:

(1) $$\frac{\partial }{\partial t}\left(\rho k\right)+\frac{\partial }{\partial x_{j}}\left(\rho ku_{j}\right)=\frac{\partial }{\partial x_{j}}\left[\left(\mu +\frac{\mu_{t}}{\sigma_{k}}\right)\frac{\partial k}{\partial x_{j}}\right]+G_{k}+G_{k}-\rho \varepsilon -Y_{M}+S_{k}$$

(2) $$\begin{array}{l} \frac{\partial \left(\rho \varepsilon \right)}{\partial t}+\frac{\partial }{\partial x_{i}}\left(\rho \varepsilon u_{j}\right)=\frac{\partial }{\partial x_{j}}\left[\left(\mu +\frac{\mu_{t}}{\sigma_{k}}\right)\frac{\partial \varepsilon }{\partial x_{j}}\right]+\rho C_{1}S_{e}+C_{1\varepsilon }\frac{\varepsilon }{K}C_{3\varepsilon }G_{b}\\ -\rho C_{2}\rho \frac{\varepsilon^{2}}{k+\sqrt{\varepsilon \nu }}+S_{\varepsilon } \end{array}$$

where,

$$C_{1}=\max\left[0.43,\frac{\eta }{\eta +5}\right],\;\eta =S\frac{k}{\varepsilon },\;S=\sqrt{2S_{ji}S_{ji}},$$

*G_k_*, and *G_b_* are the turbulent energy generation terms due to velocity gradient and buoyancy, respectively. *Y_M_* is the influence term of pulsating expansion on turbulent dissipation in compressible turbulent flow; *C*_2_ and *C*_1*ε*_ are model constants; *σ_k_* and *σ_ε_* are turbulent energy and turbulent energy dissipation Prandtl numbers, respectively; and *S_k_* and *S_ε_* are user-defined source terms.

The density-solver is selected, and the boundary conditions are set to the pressure inlet and pressure outlet, respectively. The turbulence intensity at the inlet is defined as 5%. To give reasonable results for the high Reynolds number wall bounded flow, the near-wall treatment is specified as the standard wall function. The ejector wall is set to a no-slip condition. Initial conditions: inlet pressure parameter *p*_0_ = 0.087 Mpa, and inlet temperature *T*_0_ = 390.15 K.

In this work, the spontaneous condensation of steam inside a two-dimensional nozzle is simulated using numerical simulations by introducing liquid phase mass fraction and droplet number transfer equations. The properties of steam are shown in Table 2. The effect of primary fluid parameters on spontaneous condensation is investigated, which in turn provides a basis for assessing the effect of the steam spontaneous condensation phenomenon on the overall micro-ejector flow process.

## 3. Mathematical Models

### 3.1. Conservation Equations

The Eulerian–Eulerian two-fluid model is used to describe the behavior of the wet steam flow. Since the condensation process is accompanied by mass and heat transfer between the liquid and gas phases, the mass fraction of the liquid phase, and droplet number density number transfer equations are introduced, and a set of mass, momentum, and energy conservation equations for the nucleation rate and droplet mass growth rate relations are established to close the two-phase flow.

The flow field in a steam ejector can be described as constant, compressible, steady, axial, and invariant [27,28]. The Favre-averaged Navier–Stokes equation is more applicable when the fluid concentration varies. The total energy equation for viscous dissipation combined with the gas laws was included in this analysis. The governing equation can be expressed in its compact Cartesian form as:

The continuity equation:

(3) $$\frac{\partial \rho }{\partial t}+\frac{\partial }{\partial x_{i}}\left(\rho u_{i}\right)=0$$

The momentum equation:

(4) $$\frac{\partial }{\partial t}\left(\rho u_{i}\right)+\frac{\partial }{\partial x_{j}}\left(\rho u_{i}u_{j}\right)=-\frac{\partial P}{\partial x_{i}}+\frac{\partial \tau_{ij}}{\partial x_{j}}$$

The energy equation:

(5) $$\frac{\partial }{\partial t}\left(\rho E\right)+\frac{\partial }{\partial x_{i}}\left(u_{i}\left(\rho E+P\right)\right)=\vec{\nabla }\cdot \left(\alpha_{eff}\frac{\partial T}{\partial x_{i}}\right)+\vec{\nabla }\cdot \left(u_{j}\left(\tau_{ij}\right)\right)$$

where,

(6) $$\tau_{ij}=\mu_{eff}(\frac{\partial u_{i}}{\partial x_{j}}+\frac{\partial u_{j}}{\partial x_{i}})-\frac{2}{3}\mu_{eff}\frac{\partial u_{k}}{\partial x_{k}}\delta_{ij}$$

with

(7) $$\rho =\frac{P}{RT}$$

### 3.2. Wet Steam Flow Transport Equation

The first gain transport equation that controls the mass ratio in the condensate phase can be formulated as [29,30]:

(8) $$\frac{\partial \left(\beta \rho \right)}{\partial t}+\nabla \cdot \left(\rho \beta \vec{u}\right)=\Gamma$$

Here, Γ is the rate of matter production due to condensation and volatilization. This rate is related to the nucleation rate *I* (second/number of new droplets per unit volume) and the growth/disappearance of those droplets [31,32]:

(9) $$\Gamma =\frac{4}{3}\pi \rho_{l}Ir^{*3}+4\pi \rho_{l}\eta {\bar{r}}^{2}\frac{\partial \bar{r}}{\partial t}$$

The nucleation rate [33,34] can be described in the following form:

(10) $$I=\frac{q_{c}}{\left(1+\theta \right)}\left(\frac{\rho_{v}^{2}}{\rho_{l}}\right)\sqrt{\frac{2\sigma }{M_{}^{3}\pi }}\exp(-\frac{4\pi \cdot r^{*2}\sigma }{3KT})$$

Here, *θ* is a non-isothermal correction factor [35], and it is written as:

(11) $$\theta =\frac{2(\gamma \gamma 1)}{\left(\gamma +1\right)}\left(\frac{h_{v}}{RT}\right)(\frac{h_{v}}{RT}-0.5)$$

The critical droplet radius r*, above which the droplet will grow and below which the droplet will evaporate [36], is given by:

(12) $$r^{*}=\frac{2\sigma }{\rho_{l}RT\ln S}$$

The transfer of mass to the droplets and the transfer of droplet heat to the vapor latent heat are the two mechanisms of water vapor to water vapor transfer.

Droplet size is influenced by two mechanisms: the transfer of mass from the vapor to the droplets, and the transfer of heat from the droplets to the vapor in the form of latent heat [37]. It can be written as shown below:

(13) $$\frac{\partial \bar{r}}{\partial t}=\frac{P}{h_{v}\rho_{l}\sqrt{2\pi RT}}\cdot \frac{\gamma +1}{2\gamma }C_{p}\left(T_{0}-T\right)$$

The droplet number density *η* is the number of droplets per unit volume, and it can be represented as:

(14) $$\eta =\frac{\beta }{(1-\beta )V_{d}\left(\rho_{l}/\rho_{v}\right)}$$

where, *V*_d_ is the average droplet volume, defined as:

(15) $$V_{d}=\frac{4}{3}\pi \cdot {\bar{r}}^{3}$$

### 3.3. Wet Steam State Equation 

Under the condition that the thermodynamic and transport properties of the ideal gas remain constant, the equation of state of the ideal gas is:

(16) ρ=P/(RT)

The equation of state for wet steam, is derived as follows [33]:

(17) $$P=\rho_{v}RT(1+B\rho_{v}+C\rho_{v}{}^{2})$$

where, *B* and *C* are the second and third virial coefficients, respectively, given by the following empirical expressions:

(18) $$B=a_{1}{\left(1+\frac{\tau }{\alpha }\right)}^{-1}+a_{2}e^{\tau }{\left(1-e^{-\tau }\right)}^{\frac{5}{2}}+a_{3}\tau$$

where, *τ* = 1500/*T*, *α* = 10,000, *a*_1_ = 0.0015, *a*_2_ = −0.000942, and *a*_3_ = −0.0004882.

(19) $$C=a\left(\tau -\tau_{0}\right)e^{-\alpha \tau }+b$$

where, *τ* = *T*/647.286, *τ*_0_ = 0.8978, *α* = 11.16, *a* = 1.772, and *b* = 1.5 × 10^−6^.

*C_p_* is the wet steam isobaric specific heat capacity, *h* is specific enthalpy, and *s* is specific entropy, determined using the following Equations (20)–(22) [36]:

(20) $$\begin{array}{cc} C_{p}=C_{p0}\left(T\right)+ & R(((1-\alpha_{v}T)(B-T\frac{dB}{dT})-T^{2}\frac{d^{2}B}{dT^{2}})\rho_{v}+((1-2\alpha_{v}T)C+\alpha_{v}T^{2}\frac{dC}{dT}\\ & \;-T^{2}\frac{d^{2}C}{dT^{2}}/2)\rho_{v}{}^{2}) \end{array}$$

(21) $$h=h_{0}(T)+RT((B-T\frac{dB}{dT})\rho_{v}+(C-T\frac{dC}{dt}/2)\rho_{v}{}^{2})$$

(22) $$s=s_{0}(T)+R(\ln\rho_{v}+(B+T\frac{dB}{dT})\rho_{v}+(C+T\frac{dC}{dT})/2)\rho_{v}{}^{2})$$

Here, *C_p_*_0_ is the isobaric specific heat at zero pressure, *h*_0_ is the standard state enthalpy, and *s*_0_ is the standard state entropy. 

## 4. Results and Discussion

### 4.1. Comparison of Wet Steam Simulation and Ideal Gas Simulation Results

**Figure 3:**
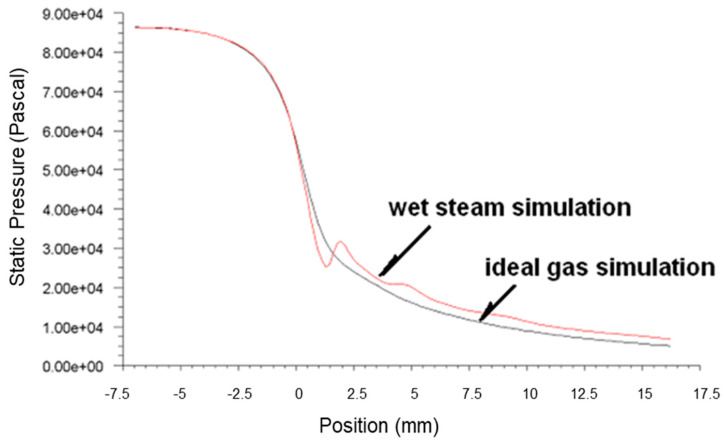
Plots of static pressure along nozzle axis based on ideal gas and wet steam simulation.

**Figure 4:**
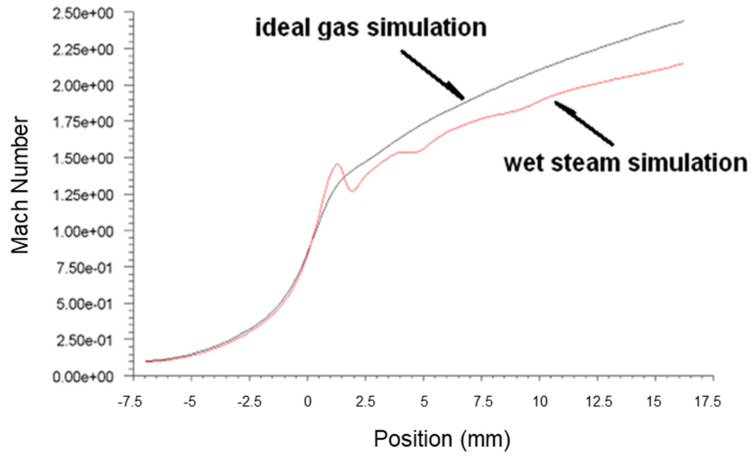
Plots of Mach number along nozzle axis based on ideal gas and wet steam simulation.

**Figure 5:**
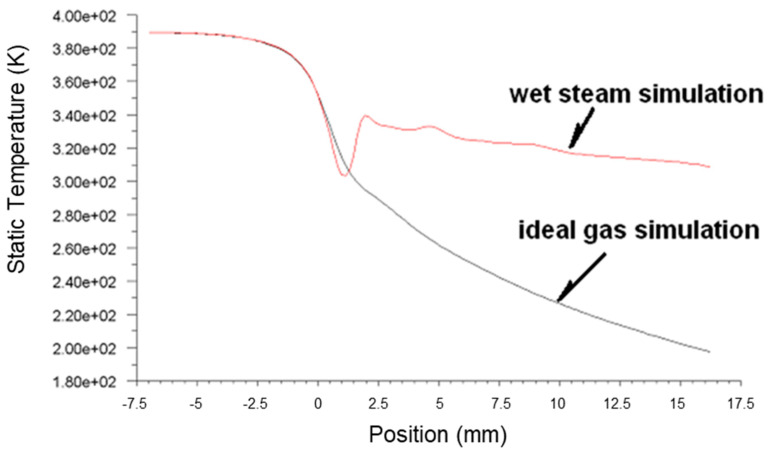
Plots of static temperature along nozzle axis based on ideal gas and wet steam simulation.

Figure 3, Figure 4 and Figure 5 present the pressure, Mach number and temperature distribution of the primary fluid at the nozzle axis under the ideal gas assumption and wet steam operational conditions, respectively.

In Figure 3, it can be seen that the pressure surged rapidly due to the sudden release of condensation heat from wet steam at about 2 mm downstream of the nozzle throat. This area where the spontaneous condensation occurred is also known as Wilson Point. The pressure continued its downward trend after the condensation impulse wave, and the pressure of the wet steam at the nozzle outlet was larger than the pressure of the ideal gas.

It was observed that the speed of the wet steam at Wilson Point experienced a sudden drop, which was relatively lower than the ideal gas, as shown in Figure 4. The difference between the two curves tends to increase gradually in the subsequent flow. The difference between the two curves at the nozzle outlet was about 9%. The formation of the condensation impulse wave due to the spontaneous condensation resulted in the sudden drop of the fluid flow at 4 mm downstream of the nozzle throat. Although the pressure curve remained rising at the subsequent flow after the impulse wave, the overall pressure is still greater than the pressure value obtained from the ideal gas simulation. Interestingly, the number of droplets formed due to the wet steam condensation was not further increased. However, the liquid phase content maintains an increasing trend [8], causing the liquid content of the fluid to increase gradually, thereby reducing the speed. The process of condensation involves the generation and accumulation of droplets. Despite the fact that the number of droplets was constant after the spontaneous condensation of fluid, the generation of droplets gradually increased the liquid content [2], thereby slowing down the rapid rise trend of the wet steam flow rate.

Figure 5 illustrates the transonic behavior of the wet steam in the nozzle after the spontaneous condensation. The temperature of fluid experienced a sudden increase due to a large amount of the latent heat of condensation formed. It was recorded that the temperature at the nozzle outlet was above 320 K, demonstrating a significant difference from the ideal gas simulation result of below 273 K. This was due to the high liquid content in the fluid, and the droplets displayed a tendency to grow after the condensation impulse wave resulted in a reduction in the flow rate of the fluid. The overall inflationary trend was reduced, thereby slowing the declining trend of temperature. The temperature of fluid after the condensation impulse wave was acquired from the wet steam simulation remained higher than the temperature obtained from the ideal gas simulation, with a significant difference of 34% at the nozzle outlet.

Based on the above simulation results, the spontaneous condensation of fluid that occurred in the nozzle demonstrated a significant impact on the flow state of the fluid: the latent heat of condensation released by the spontaneous condensation increased the fluid pressure and reduced the flow rate in the nozzle. The increase in the fluid pressure and the reduction in the flow rate at the nozzle outlet would reduce the ejection efficiency, which caused negative effects on the ejection of the secondary fluid. It was found that the results obtained from the ideal gas simulation displayed a significant difference from the actual flow. Hence, the use of the wet steam model demonstrates a more realistic reflection of the flow behavior in the nozzle.

### 4.2. The Effects of Steam Parameters on Spontaneous Condensation

#### The Effects of Primary Fluid Pressure on the Spontaneous Condensation Flow of Fluid

**Figure 6:**
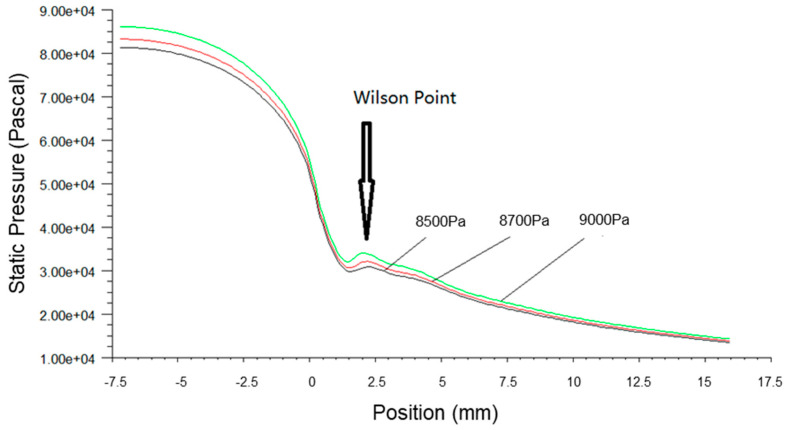
A plot of static pressure along nozzle axis at different inlet pressures.

**Figure 7:**
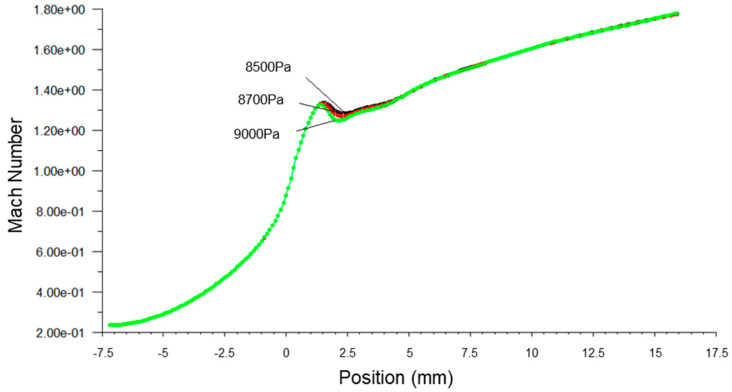
A plot of static temperature along nozzle axis at different inlet pressures.

**Figure 8:**
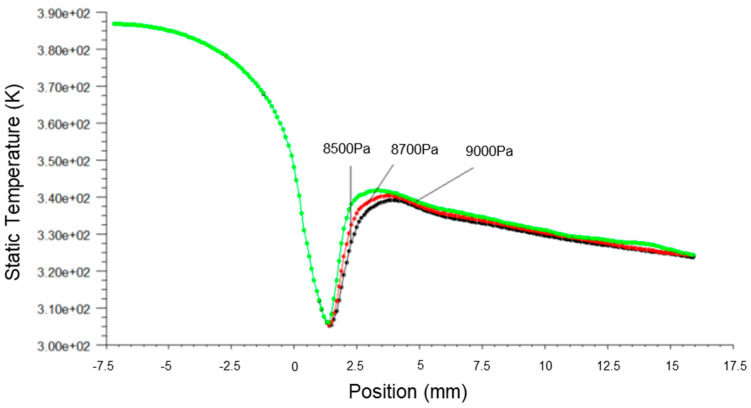
A plot of Mach number along nozzle axis at different inlet pressures.

Figure 6, Figure 7 and Figure 8 present the fluid pressure in the nozzle, Mach number and temperature distribution, respectively, at different inlet fluid pressures.

The static pressure of the fluid along the axial direction was increased with the inlet fluid pressure, and the Wilson Point moved towards the nozzle throat, as shown in Figure 6. This accounted for the increase in energy content in wet steam due to the high pressure at the nozzle inlet that caused the earlier release of latent heat of condensation. Consequently, this led to the sudden rise of pressure, the increase in the mass fraction of the liquid phase, the increase in latent heat of condensation by the fluid per unit of time and enhanced the heating effects on fluid flow. The pressure fluctuations at the Wilson Points and the increment rate of fluid pressure also increased correspondingly.

It can be seen from Figure 7 that the Mach number along the axial direction, which was close to the Wilson Point, changed subject to the inlet fluid pressure. The increase in fluid inlet pressure reduced the Mach number near the Wilson Point, but the remaining distributed Mach number was constant. This finding indicated that the primary fluid passing through the nozzle was able to obtain a large flow rate when the fluid pressure was larger than the stabilized working pressure.

Figure 8 shows that the temperature distribution under different inlet fluid pressures remained unchanged before the spontaneous condensation. In contrast, the axial temperature close to the Wilson Point increased with the inlet fluid pressure, shifting the maximum value forward. The temperature distribution converged after the Wilson Point.

**Figure 9:**
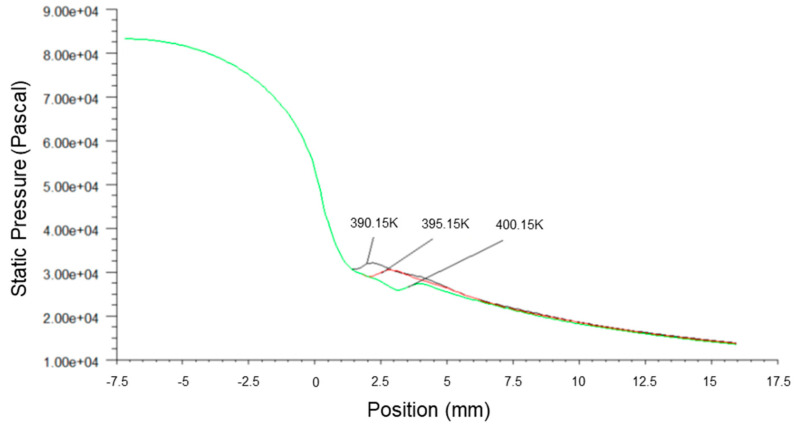
A plot of static pressure along nozzle axis at different inlet temperatures.

**Figure 10:**
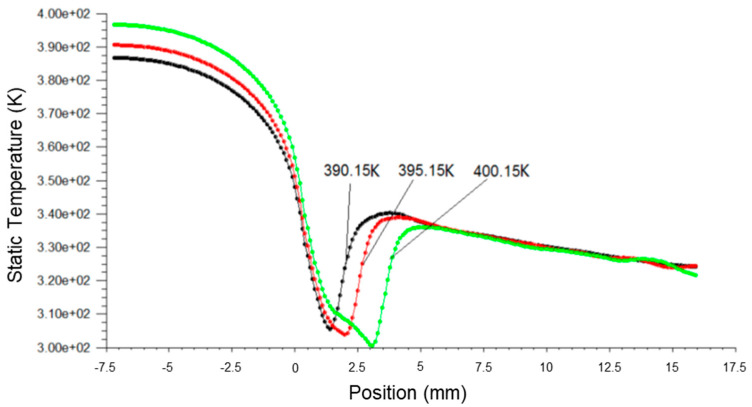
A plot of Mach number along nozzle axis at different inlet temperatures.

**Figure 11:**
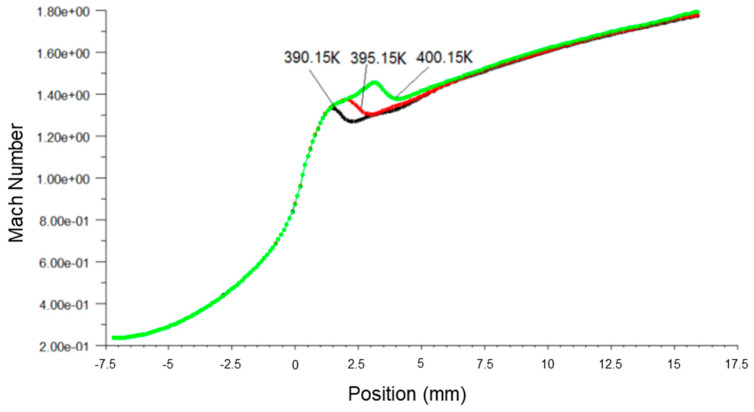
A plot of static temperature along the nozzle axis at different inlet temperatures.

Figure 9, Figure 10 and Figure 11 present the pressure, Mach number and temperature distribution of fluid flow, respectively, at different inlet temperatures.

Based on Figure 9, it was observed that when the primary fluid inlet temperature was increased, the spontaneous condensation point (Wilson Point) in the ejector gradually moved downstream, where the distance away from the nozzle throat was increased from 1.6 mm to 4 mm. The pressure step order decreased correspondingly with the Wilson Point, thereby effectively inhibiting spontaneous condensation and energy loss, and hence improving the ejection efficiency.

Figure 10 shows that the Mach number increased with the rise of inlet temperature, which maximizes the downstream movement of the Wilson Point. The high fluid inlet temperature possessed high work capacity, thereby facilitating efficient ejection of the secondary fluid.

The increase in the fluid inlet temperature resulted in an increment in the fluid flow temperature in the ejector, as shown in Figure 11. The rise in temperature of the fluid delayed the onset of condensation and the release of latent heat of condensation. The highest temperature step over position moved downstream from x = 3 mm to x = 4.5 mm, and the final outlet temperature converged. The phenomenon of the spontaneous condensation that occurred in the nozzle reduced the possibility of the low temperature or icing issues occurring due to excessive inflation of fluids, thereby achieving stabilized operational conditions of the steam ejector.

## 5. Conclusions

In this paper, various aspects of numerical modeling, numerical methods, nucleation, and droplet growth theory for the numerical study of the two-phase nonequilibrium condensing flow of wet steam are comprehensively analyzed and discussed. Combining droplet nucleation and growth theory, a set of two-phase flow equations for the existence of spontaneous condensing wet steam is described under the assumption of no slip. The two-phase flow is numerically simulated for the spontaneous condensation of steam in the nozzle under nonequilibrium conditions. The distribution of the two-phase parameters of steam along the nozzle axial direction is obtained. The influence of the inlet pressure and inlet temperature of the primary fluid on the condensation flow in the nozzle under transonic flow conditions was analyzed via numerical calculations of the two-phase condensation flow of wet steam. A theoretical basis is provided to improve the overall efficiency and operational safety of the ejector, as well as to guide the design of the through-flow section. Detailed findings are as follows:

- The numerical model that considered the spontaneous condensation of transonic wet steam flow was established. The spontaneous condensation was numerically simulated.
- The spontaneous condensation of primary fluid released the latent heat of condensation, leading to the sudden rise and drop in fluid pressure and flow rate, respectively. Compared to the ideal gas assumption simulation results, the higher outlet wet steam pressure and lower flow rate negatively affect the ejection of the secondary fluid.
- The effects of the primary fluid inlet pressure and temperature on the transonic flow with the spontaneous condensation process in the nozzle were numerically investigated. The results indicated that the parametric of primary fluids directly impacts the spontaneous flow of the transonic fluid flow in the ejector. The effects of the operating parameters on the flow of fluid should be taken into account during the design of the nozzle to ensure the operating performance and stability of the fluid flow.
- The condensation position can be advanced by keeping the total inlet temperature of the primary fluid constant and increasing the total inlet pressure. Condensation occurs downstream of the throat, and the closer the condensation position is to the throat, the more intense the pressure impulse caused by condensation and the more significant the sudden change in pressure.

## Figures and Tables

**Table 1:** The dimensions of the components.

Component	Size (mm)
Nozzle Throat Diameter	2.5
Nozzle Inlet Diameter	8.25
Inlet Expansion Angle	22°
Outlet Expansion Angle	18°
Total Length of nozzle	25

**Table 2:** Steam properties.

Properties	Value
Dynamic viscosity	1.34 × 10^−5^ kg·m^−1^·s^−1^
Thermal conductivity	0.00261 W·m^−1^·K^−1^
Specific heat capacity	2014.00 J·kg^−1^·K^−1^
Molecular weight	18.01534 kg·kmol^−1^

## Data Availability

The research data supporting this publication are provided within this paper.

## Nomenclature

*ρ, ρ_l_, ρ_v_*	density, liquid density, vapor density (kg/m^3^)	*M*	molecular mass (kg)
*u*	velocity (m/s)	*γ*	specific heat capacities ratio
*U′*	fluctuation velocity (m/s)	*h_v_*	vapor specific enthalpy (J/kg)
*P*	pressure (Pa)	*R*	gas-law constant
*τ_ij_*	stress tensor	*S*	super saturation ratio
*E*	total energy	*C_p_*	isobaric heat capacity (J/(kgK))
*α_eff_*	effective thermal conductivity	*T*_0_	droplet temperature (K)
*β*	mass fraction	*V_d_*	average droplet volume (m^3^)
Γ	mass generation rate (kg/s)	*B, C*	Virial coefficients (m^3^/kg, m^6^/kg^2^)
$\bar{r}$	droplet average radius (m)	*α_v_*	volume fraction
*r**	critical droplet radius (m)	*h*	specific enthalpy (J/kg)
*I*	nucleation rate (1/s)	*s*	specific entropy (J/(kg·mol·K))
*η*	droplet number density (1/m^3^)	*k*	turbulent kinetic energy
*q_c_*	evaporation coefficient	*μ_t_*	eddy viscosity
*θ*	non-isothermal correction factor	*ε*	turbulence kinetic energy dissipation
*σ*	droplet surface tension (kg/m)	*S_ij_*	strain rate
*T*	thermal temperature (K)	*C*_2_, *C*_1*ε*_, *C*_3*ε*_, *σ_k_*, *σ_ε_*	model coefficients
*K*	Boltzmann constant	*S_k_, S_ε_*	source terms
